# Assessment of alveolar bone loss in diabetic patients with class II composite resin restorations

**DOI:** 10.6026/9732063002001485

**Published:** 2024-11-05

**Authors:** Abdo Mohammed Mohammed Abdulrazzaq

**Affiliations:** 1 Department of Preventive Dental Science, Faculty of Dentistry, Najran University, Najran, Saudi Arabia

**Keywords:** Class II composite restorations, alveolar bone loss, plaque index, gingival index, diabetic, periodontal health

## Abstract

This study evaluated the impact of Class II composite resin restorations on alveolar bone loss in diabetic patients by comparing those with restorations to a control group without such treatments at the Faculty of Dentistry, Najran University, Saudi Arabia. A total of 64 diabetic patients were divided into two groups: control group G1 (n=32) with no composite resin restorations and test group G2 (n=32) with composite resin restorations. Metrics assessed included plaque index (P.I), gingival index (G.I), HbA1c levels, and alveolar bone loss percentage. Results showed that test group G2 had higher mean values for P.I (2.73 vs. 2.51), G.I (2.53 vs. 2.22), and alveolar bone loss (48.28% vs. 44.16%) compared to control group G1. HbA1c levels were slightly lower in G2 but not significantly different. Significant differences were found in P.I, G.I, and alveolar bone loss between the groups (p-values: 2.767e-06, 2.895e-13, and 3.585e-08, respectively). Diabetic patients with composite resin restorations exhibited poorer oral hygiene, greater gingival inflammation, and increased alveolar bone loss. Enhanced preventive care and monitoring are recommended for these patients. However, clinically, this highlights the importance of careful material selection, enhanced periodontal monitoring, and tailored preventive care to prevent further bone loss and maintain oral health in diabetic patients.

## Background:

Diabetes mellitus has a well-established impact on oral health, contributing to an increased risk of periodontal diseases and subsequent alveolar bone loss [1]. Patients with diabetes often exhibit more severe forms of periodontal disease, which is exacerbated by poor glycemic control and the presence of dental restorations [2]. Composite resin restorations are effective for dental repair but can impact plaque accumulation and gingival health; this is especially concerning for diabetic patients, who are at a higher risk for periodontal complications [3]. The effect of dental restorations on alveolar bone loss is complex, intriguing and it is a key subject in periodontal health, dental restorations, such as fillings, crowns, implants, dentures, bridges and orthodontic appliances, have consistently demonstrated the ability to preserve alveolar bone by mimicking natural tooth structures and providing functional loading that stimulates the surrounding bone [4]. Therefore, this study aimed to fill this gap by comparing periodontal health indicators and alveolar bone loss between diabetic patients with and without class II composite resin restorations.

## Materials and Methods:

The researchers obtained ethical approval from the University's Research Ethics Committee, following all requirements. Clinical examinations and evaluations involving humans adhered to the ethical standards of the institutional/national research committee and the Helsinki Declaration (amended by the 64th WMA General Assembly, Fortaleza, Brazil, 2013) and its subsequent amendments or comparable standards.

## Participant groups and data collection:

In this observational comparative cross-sectional study, we selected 64 type 2 diabetic patients from a sample of 693 at the Faculty of Dentistry, Najran University, Saudi Arabia. The patients were divided into two groups of 32, aged 40-60 years. Control Group G1 included diabetic patients without Class II composite resin restorations, while Test Group G2 included those with such restorations. The restorations had a service life of 5 to 7 years. Clinical examinations included plaque index (PI) to determine oral hygiene status [5], gingival index (GI) to measure gingival inflammation [6]. Alveolar Bone Loss using radiographic analysis to quantify bone loss percentage [7], for each group by 2 clinicians and recorded HbA1c Levels to assess long-term blood glucose controls [8].

## Inclusion:

The inclusion criteria for selecting the control group G1 of diabetic patients were:

[1] Patients with type 2 diabetes ≥ 2 years.

[2] HbA1c levels ≥ 7%.

[3] 40 - 60 years of age

[4] A minimum of 10 teeth remaining.

[5] Without Class II composite resin restorations.

The inclusion criteria for selecting the test group G2 of diabetic patients were:

[1] Patients with type 2 diabetes ≥ 2 years.

[2] HbA1c levels ≥ 7%.

[3] 40 - 60 years of age

[4] A minimum of 10 teeth remaining.

[5] Class II composite resin restorations life range 5 - 7 years.

## Exclusion criteria:

Patients with severe systemic diseases unrelated to diabetes or those who had received periodontal treatment within the previous six months were excluded from the study. This was done to eliminate confounding factors and ensure that the focus remained on the impact of diabetes and restorative materials on periodontal health.

## Results:

A total of 64 participants were enrolled in this study, with an equal distribution of 32 (50%) diabetic patients in each group. Control Group 1 (G1) consisted of diabetic patients without Class II composite resin restorations, while Test Group 2 (G2) included diabetic patients with these restorations. The study aimed to compare the periodontal health of G1 and G2 patients. Data were statistically analysed using SPSS version 18 software. Two statistical tests were employed: Descriptive Statistics and Two-Sample T-Test. The results for both groups were analysed, and the variations between them were found to be statistically significant.

## Analysis:

The study reported the following key findings: The mean Plaque Index (P.I) was 2.51 (SD = 0.18) with values ranging from 2.25 to 2.75. The mean Gingival Index (G.I) was 2.22 (SD = 0.04) with values ranging from 2.00 to 2.25. The mean HbA1c level was 8.82% (SD = 0.31), with a range of 8.50% to 9.40%. The mean percentage of bone loss was 44.16% (SD = 3.25), with a range from 39.00% to 50.00% (Table 1).

## Analysis:

The study found the following: The mean Plaque Index (P.I) was 2.73 (SD = 0.16), with values ranging from 2.25 to 3.00. The mean Gingival Index (G.I) was 2.53 (SD = 0.18), with a range of 2.20 to 2.80. The mean HbA1c level was 8.70% (SD = 0.41), ranging from 8.10% to 9.40%. The mean bone loss percentage was 48.28% (SD = 1.78), with values ranging from 45.00% to 52.00% (Table 2).

## Interpretation of comparison:

The study found significant differences between the groups: Test Group G2 had a higher Plaque Index (2.73) than Control Group G1 (2.51), indicating poorer oral hygiene (p=2.767e-06). Gingival Index was also higher in G2 (2.53) compared to G1 (2.22), showing increased gingival inflammation (p=2.895e-13). No significant difference in HbA1c levels was observed (p=0.210). However, G2 experienced significantly greater bone loss (48.28%) than G1 (44.16%) (p=3.585e-08).

## Analysis:

**Figure 1:**
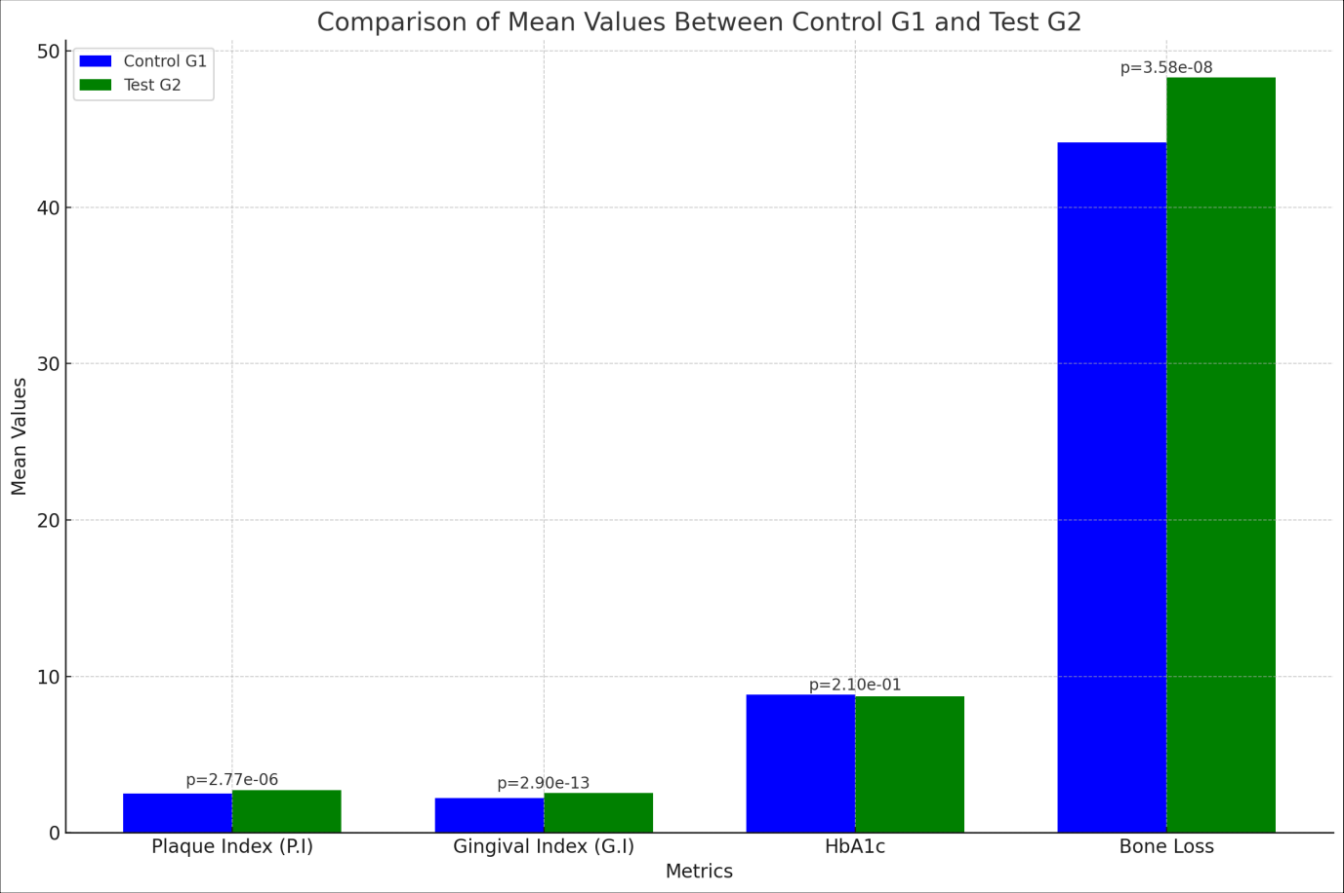
Comparison of mean values between Control Group G1 and Test Group G2

The study found that Test Group G2 had a higher mean Plaque Index (P.I) of 2.73 compared to Control Group G1's 2.51, indicating worse oral hygiene in G2 (t-statistic: -5.159, p-value: 2.767e-06). Similarly, the Gingival Index (G.I) was higher in G2 at 2.53, compared to 2.22 in G1, reflecting greater gingival inflammation (t-statistic: -9.239, p-value: 2.895e-13). No significant difference was observed in HbA1c levels between G1 (8.82) and G2 (8.70) (t-statistic: 1.265, p-value: 0.210). However, G2 experienced greater mean bone loss at 48.28% compared to G1's 44.16% (t-statistic: -6.290, p-value: 3.585e-08) (Table 3 and Figure 1).

## Interpretation of comparison:

The study reveals that Test Group G2 has significantly higher Plaque and Gingival Indexes compared to Control Group G1, indicating worse oral hygiene and more gingival inflammation in G2. The very low p-values confirm that these differences are statistically significant. However, no significant difference was observed in HbA1c levels between the two groups, suggesting similar blood glucose control. Additionally, Test Group G2 exhibits significantly higher bone loss than Control Group G1, with the low p-value confirming the statistical significance of this difference, indicating more alveolar bone loss in G2. These visualizations highlight the significant differences in periodontal health and alveolar bone loss between the two groups, emphasizing the need for careful monitoring and preventive measures for patients receiving class II composite resin restorations.

## Discussion:

The analysis demonstrates that diabetic patients with class II composite resin restorations exhibit significantly worse oral hygiene, increased gingival inflammation, and greater alveolar bone loss compared to those without such restorations. This is consistent with previous research highlighting the negative impact of restorative materials on periodontal health, particularly in diabetic patients [9]. The findings support earlier studies suggesting that restorative materials can exacerbate periodontal issues by increasing plaque accumulation and inflammation [10]. The lack of significant difference in HbA1c levels between the two groups indicates that the observed periodontal complications are more closely associated with the restorative treatments rather than overall glucose control [11]. This suggests that while glycemic control remains important, the mechanical and biofilm-retentive properties of restorative materials may play a more direct role in periodontal health. These results underscore the necessity for rigorous preventive care and regular monitoring for diabetic patients undergoing restorative treatments. Enhanced oral hygiene practices and preventive measures should be emphasized to mitigate the potential adverse effects of dental restorations [12]. However, the study's limitations include a small sample size, short follow-up period, and lack of data on smoking, obesity, and restoration quality (*e.g.*, smoothness and proximal overhangs). These factors may affect the generalizability and understanding of findings. Future research with larger samples, extended follow-up, and consideration of these variables could provide more comprehensive insights into the long-term effects of restorative materials on periodontal health in diabetic patients [13].

## Conclusion:

Diabetic patients who receive Class II composite resin restorations demonstrate significantly higher Plaque Index, Gingival Index, and alveolar bone loss compared to diabetic patients who do not undergo such restorations. These findings highlight the critical importance of maintaining meticulous oral hygiene and ensuring regular periodontal monitoring for diabetic patients undergoing restorative dental treatments. The increased risk of periodontal complications associated with composite resin restorations in this population necessitates a proactive approach to managing oral health, emphasizing preventive care and early intervention to mitigate the potential for adverse outcomes.

## Figures and Tables

**Table 1:** Descriptive Statistics for Control Group 1 (G1)

**Statistic**	**Age**	**P.I**	**G.I**	**HbA1c**	**Bone Loss %**
Count	32	32	32	32	32
Mean	50.53	2.51	2.22	8.82	44.16
Std.Dev	4.29	0.18	0.04	0.31	3.25
Min	45	2.25	2	8.5	39
25th Pctl	47.75	2.44	2.2	8.6	42
Median	49	2.5	2.22	8.7	43
75th Pctl	53.5	2.75	2.25	8.9	46.25
Max	60	2.75	2.25	9.4	50

**Table 2:** Descriptive statistics for test group 2 (G2)

**Statistic**	**Age**	**P.I**	**G.I**	**HbA1c**	**Bone Loss %**
Count	32	32	32	32	32
Mean	50.03	2.73	2.53	8.7	48.28
Std.Dev	3.63	0.16	0.18	0.41	1.78
Min	45	2.25	2.2	8.1	45
25th Pctl	47.75	2.6	2.4	8.5	47
Median	49	2.75	2.5	8.6	48
75th Pctl	51.5	2.8	2.7	8.9	49
Max	59	3	2.8	9.4	52

**Table 3:** Two-sample T-test results for control group 1 (G1) and Test Group 2 (G2)

**Metric**	**Control G1 Mean**	**Test G2 Mean**	**t-statistic**	**p-value**
Plaque Index (P.I)	2.51	2.73	-5.159	2.77E-06
Gingival Index (G.I)	2.22	2.53	-9.239	2.90E-13
HbA1c	8.82	8.7	1.265	0.21
Bone Loss	44.16	48.28	-6.29	3.59E-08
